# Remission in Crohn’s disease is accompanied by alterations in the gut microbiota and mucins production

**DOI:** 10.1038/s41598-019-49893-5

**Published:** 2019-09-13

**Authors:** Daniéla Oliveira Magro, Andrey Santos, Dioze Guadagnini, Flavia Moreira de Godoy, Sylvia Helena Monteiro Silva, Wilson José Fernandes Lemos, Nicola Vitulo, Sandra Torriani, Lilian Vital Pinheiro, Carlos Augusto Real Martinez, Mario José Abdalla Saad, Claudio Saddy Rodrigues Coy

**Affiliations:** 1 Department of Internal Medicine, FCM, State University of Campinas-UNICAMP, Campinas, SP Brazil; 2 Department of Surgery, FCM, State University of Campinas-UNICAMP, Campinas, SP Brazil; 3 Pontifical Catholic University of Campinas, Campinas, SP Brazil; 4 Department of Biotechnology, University of Verona, Verona, Italy

**Keywords:** Dysbiosis, Crohn's disease

## Abstract

Previous studies have demonstrated that patients with Crohn’s disease (CD) in remission do not exhibit an improvement in gut microbiota composition, which might trigger relapses. The present study investigated the dysbiosis and mucins production in CD patients during remission. We performed an analytical cross-sectional single center study, which recruited 18 CD patients and 18 healthy controls (CG) residing in the same home, meaning that all of the participants experienced the same environmental factors, with similar hygiene status, diet, pollution and other common lifestyle characteristics that may influence the composition of the gut microbiota. When compared to healthy controls, the CD patients exhibited lower microbial α-diversity (p = 0.047), a greater abundance of the Proteobacteria phylum (p = 0.037) and a reduction in the Deltaproteobacteria class (p = 0.0006). There was also a reduction in the *Akkermansia* (p = 0.002) and *Oscillospira* (p = 0.024) genera and in the proportion of the yeast *Saccharomyces cerevisiae* (p = 0.01). Additionally, CD patients in remission presented increased neutral (p = 0.001) and acid mucin (p = 0.002) concentrations. The reductions in the proportions of *Oscollospira* and *Akkermansia* genera, sulfate-reducing bacteria and *Saccharomyces cerevisiae*, observed in the CD group, may account for the increased mucins production observed in these patients.

## Introduction

Crohn’s disease (CD) is characterized by chronic inflammation of the gastrointestinal (GI) tract, and is associated with an increase in the production of inflammatory cytokines, such as interleukins (IL) and tumor necrosis factor alpha (TNF-α)^1^. The disease involves complex interactions among the host immune system, intestinal mucosa and gut microbiota. Interestingly, the gut microbiome has been receiving more attention, and is thought to play a more important role than previously thought^2^. For example, alterations in microbial composition of the intestines, or dysbiosis, and damage to the intestinal mucosal barrier can lead to frequent clinical manifestations, such as diarrhea and weight loss^3,4^. The severity of the intestinal inflammation has been associated with the largest number of colonization sites of gut microbiota with lipopolysaccharide (LPS) endotoxin activity^3–5^.

The colonic mucus barrier is considered the first line of defense against antigens and bacteria present in the intestinal lumen. It is composed of glycoproteins, trefoil factors and mucins^6^. In fact, it was previously reported that changes in the secretion patterns of mucins may be a primary event in CD or secondary to the observed inflammation^7^. Two types of mucins produced by the GI tract include, neutral and acid. In the upper GI tract, neutral mucins are predominantly secreted, while acid mucins prevail in the colon^5^. Additionally, studies have demonstrated that mucins content and expression are important for modulating short chain fatty acid (SCFA) synthesis, affecting the anti-inflammatory and immunological roles of these compounds^3,4^. Despite the apparent importance of mucins in the GI tract, the role of these proteins during the CD remission phase has not yet been deeply investigated.

Several studies, over the last 10 years, have shown that dysbiosis occurs in Inflammatory Bowel Diseases (IBD)^8–12^. More specifically, in IBD, the global composition of the gut microbiota contains specific pathogens that may be relevant to the etiology and pathogenesis of the disease. With regards to the gut microbiota of CD patients, it has been reported that there is an overall reduction in microbial diversity^13^, evidenced by alterations in the relative abundance of specific bacterial taxa^6^ and fungal communities, when compared to healthy individuals^14^.

Predominant changes described in literature regarding the gut microbiota composition of individuals with IBD and CD, include: alterations in the proportion of *Bacteroides* and *Firmicutes*, an increase in the percentage of *Gammaproteobacteria*^15,16^ and *Enterobacteriales* (i.e. *Escherichia coli* in CD with ileal disease), and a decrease in *Clostridiales* (i.e. *Faecalibacterium prausnitzii*)^17^. Furthermore, it has been observed that the amount of the fungi *Candida albicans* is increased in patients with CD^18^, whereas *Saccharomyces cerevisiae* is more abundant in non-inflamed mucosa^18^.

In CD, there are frequent relapses after periods of remission which are not entirely well understood. In fact, it is plausible that clinical remission is not accompanied by a reestablishment in the microbial balance of the GI tract, which might trigger future relapses. It is important to point out that there are no previous studies have evaluated of the gut microbiota and mucins production during CD remission. Therefore, the present study sought to compare and contrast the GI tract microbiomes and mucin expression patterns in CD patients during remission and healthy controls.

## Results

### Study population characteristics

Between June 2016 and May 2017, 36 subjects were studied, 18 patients with CD and 18 healthy controls. The subjects in the control group (CG) resided in the same house to the corresponding CD patient, sharing the same environmental status, like hygiene habitus, diet, pollution and other factors. The baseline characteristics of groups are show in detail in Table 1. According to Montreal classification, A2, L2 and B1 phenotypes were the commonest. Perianal involvement was present in 44.4%. and endoscopic remission were present in 72.3% of the CD patients. Regarding CG, one individual used probiotic for seven days, between 2–3 months prior to study.

**Table 1:** Baseline Characteristics.

Variable		CD n = 18	CG n = 18	*P* Value
**Sex No. (%)**				
Male		8 (44.4)	6 (33.3)	0.78
Female		10 (55.6)	12 (66.7)	0.83
Age (IQR) yr		42.0 (23.0–54.2)	51.0 (44.7–60.0)	0.04
Body mass index (Kg/m^2^) (IQR)		21.53 (20.0–25.5)	27.3 (21.7–31.3)	0.01
Probiotic use, No. (%)		0 (0)	0 (0)	1
CDAI (Mean ± SD)		51.03 ± 38.44	—	—
Endoscopic				—
Remission (%)		72.3	—	—
Mild-moderate (%)		27.7	—	—
Normal (%)		—	100.0	
**Smoking Status, No. (%)**				
Never		15 (83.3)	11 (61.1)	0.85
Ex		1 (5.6)	4 (22.2)	0.37
Current		2 (11.1)	3 (16.7)	1.00
No. cigarette/day (IQR)		0.0 (0.0–0.0)	0.0 (0.0–0.0)	0.67
Disease duration, year (IQR)		9.0 (3.75–18.5)	—	—
**Age at diagnosis, No (%)**	A1 <16 y	3 (16.7)	—	—
	A2 17–40 y	11 (61.1)	—	—
	A3 >40 y	3 (16.7)	—	—
**Disease location, No (%)**	L1 Ileum	5 (27.8)	—	—
	L2 Colon	9 (50.0)	—	—
	L3 Ileum-colon	3 (16.7)	—	—
**Disease behavior, No (%)**	B1 Inflammatory	11 (61.1)	—	—
	B2 Stricturing	4 (22.2)	—	—
	B3 Penetrating	2 (11.1)	—	—
**Perianal disease, No (%)**		8 (44.4)	—	—
Previous steroids, No (%)		2 (11.1)	—	—
**Medication use, No (%)**				
Mesalamine-sulfazalazine		5 (13.9)	—	—
Immunosuppressants		13 (36.1)	—	—
Anti-TNF		12 (66.7)	—	—
Antibiotics		0	0	1
History of surgery, No (%)		9 (50.0)	—	—
**Type of surgery, No (%)**				
Ileocolectomy		4 (44.4)	—	—
Colon resection		1 (11.1)	—	—
Fistula		3 (33.4)	—	—
Stenosis		1 (11.1)	—	—

Comparison between groups were performed; the χ^2^ test was used to categorical variables; Values are presented as median (IQR – interquartile range); Mann-Whitney U-test; χ^2^.

### Histological and histochemical analysis

**Figure 1:**
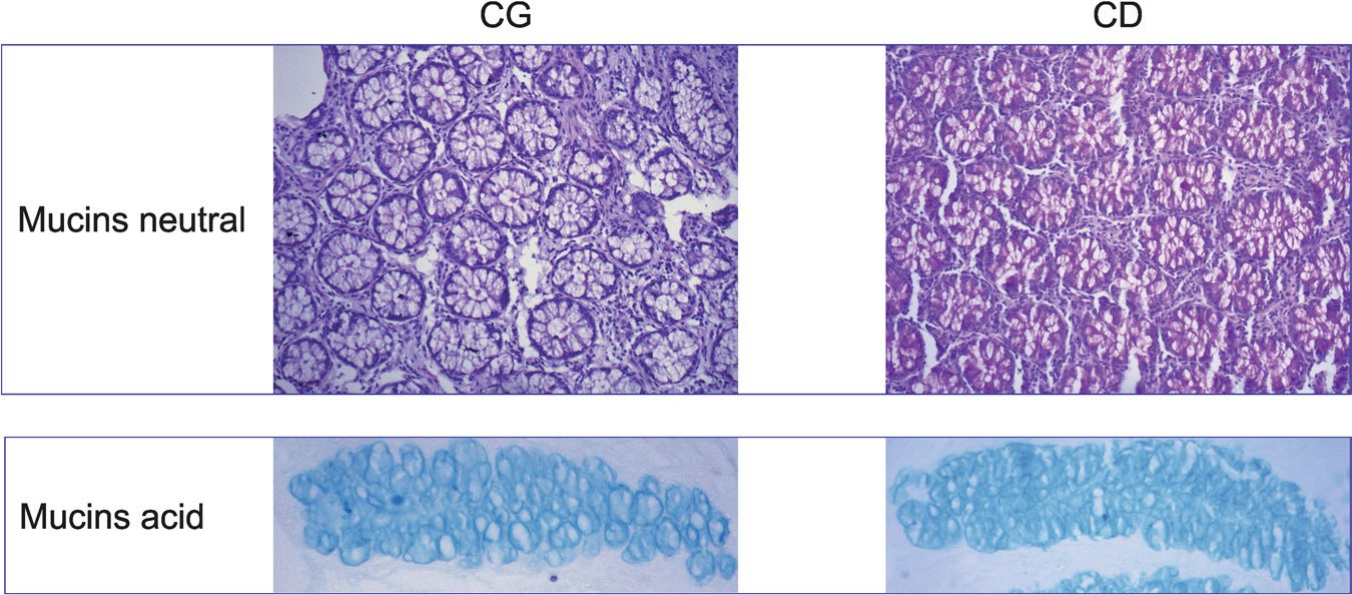
The histochemical image from neutral and acid mucins. Both mucins concentrations were increased in CD group (Mucins neutral CG *vs* CD p = 0.001; and mucin acid CG *vs* CD p = 0.002). (**A**) Neutral mucin normal from CG. (**B**) Neutral mucin of inactive CD. (**C**) Acid mucin normal from CG. (**D**) Acid mucin of inactive CD. (Mucins neutral CG *vs* CD p = 0.001; and mucin acid CG *vs* CD p = 0.002).

Both mucins concentrations were increased in CD group. Mucins neutral was 39.62 CD *vs* 33.01 CG (p = 0.001) and mucin acid was 46.03 CD *vs* 39.62 CG (p = 0.002) (Fig. 1).

### Microbial patterns in Crohn’s disease group and control group

To evaluate the gut microbiota diversity between CD and CG groups, PERMANOVA analysis was carried out. The results showed that the distribution and abundances of two groups (Supplementary Table 1S), are different (p < 0.01)

**Figure 2:**
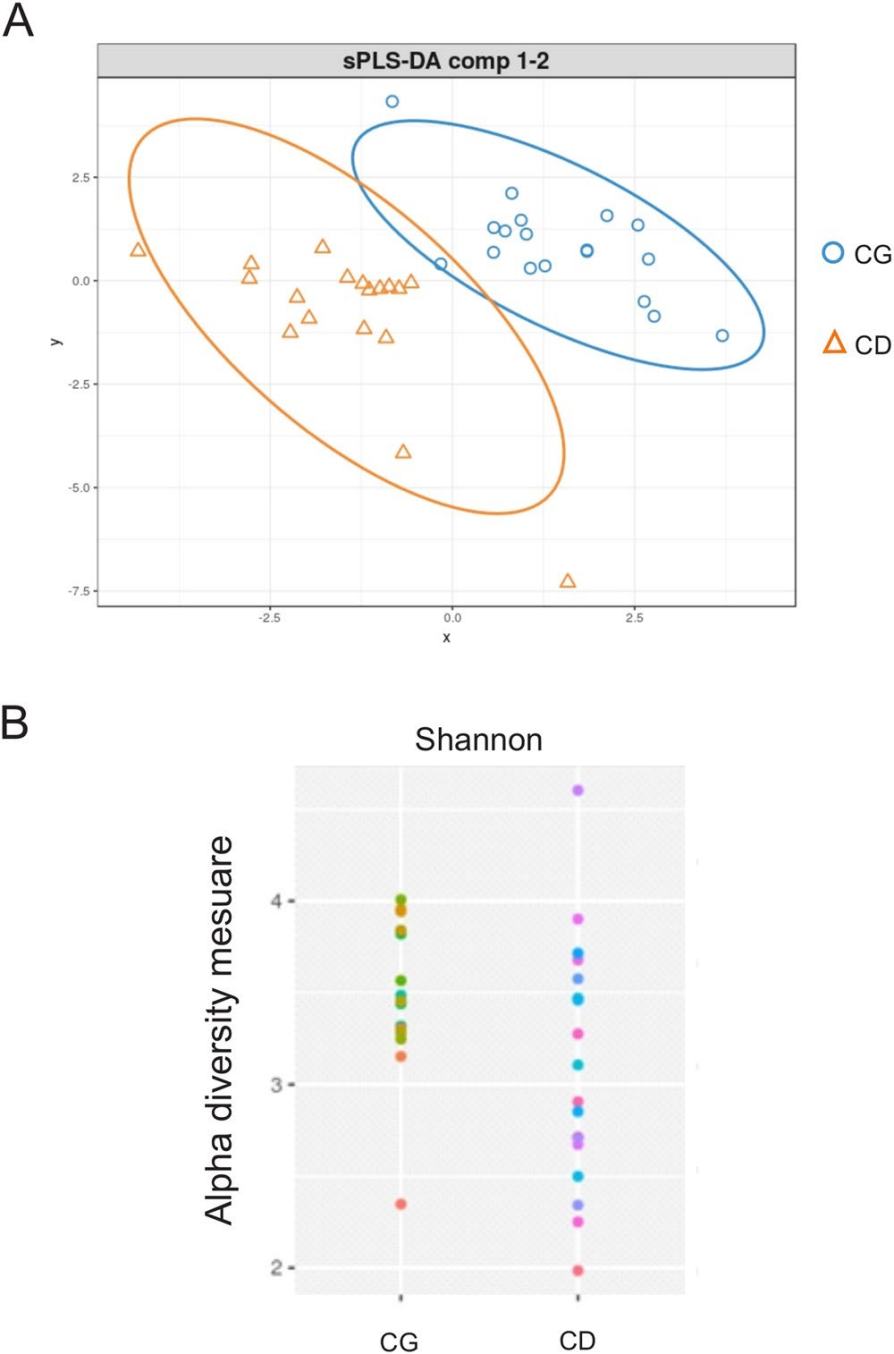
Microbial patterns in Crohn’s disease group and control group. (**A**) PLS-DA scores plot for the genera shows a clear differentiation between CG and CD groups. (**B**) Comparison of α diversity by Shannon indexes between. Each color point corresponds α diversity of one subject. CD showed a lower microbial α diversity compared with the CG (p = 0.047).

**Figure 3:**
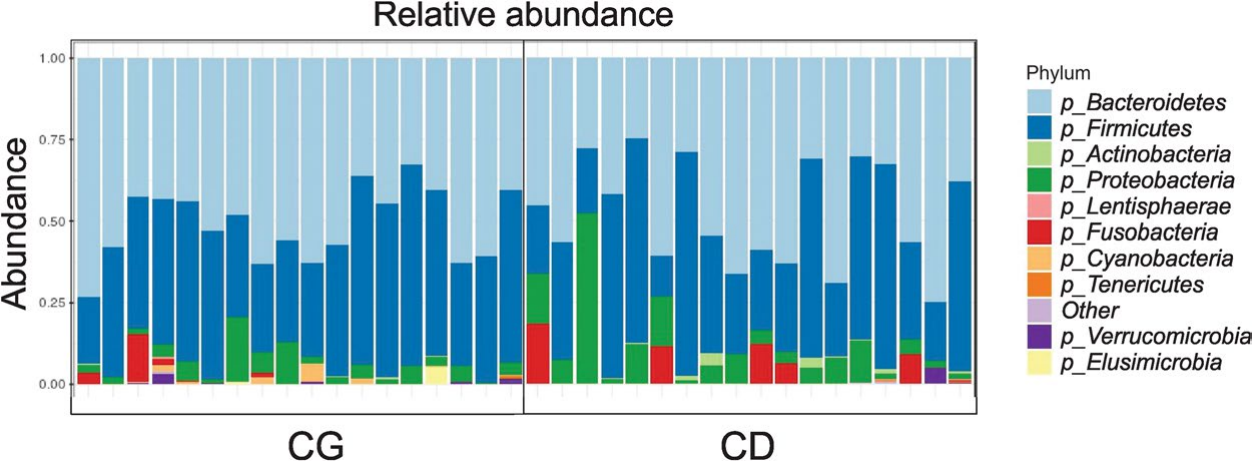
The relative abundance of bacterial phyla. Comparison of metagenomics analysis of bacterial phyla from gut microbiota in CG (n = 18 subject) and CD (n = 18 subject). The data obtained from sequencing of the hyper-variable region (V3–V4) of the bacterial 16S rRNA gene.

**Figure 4:**
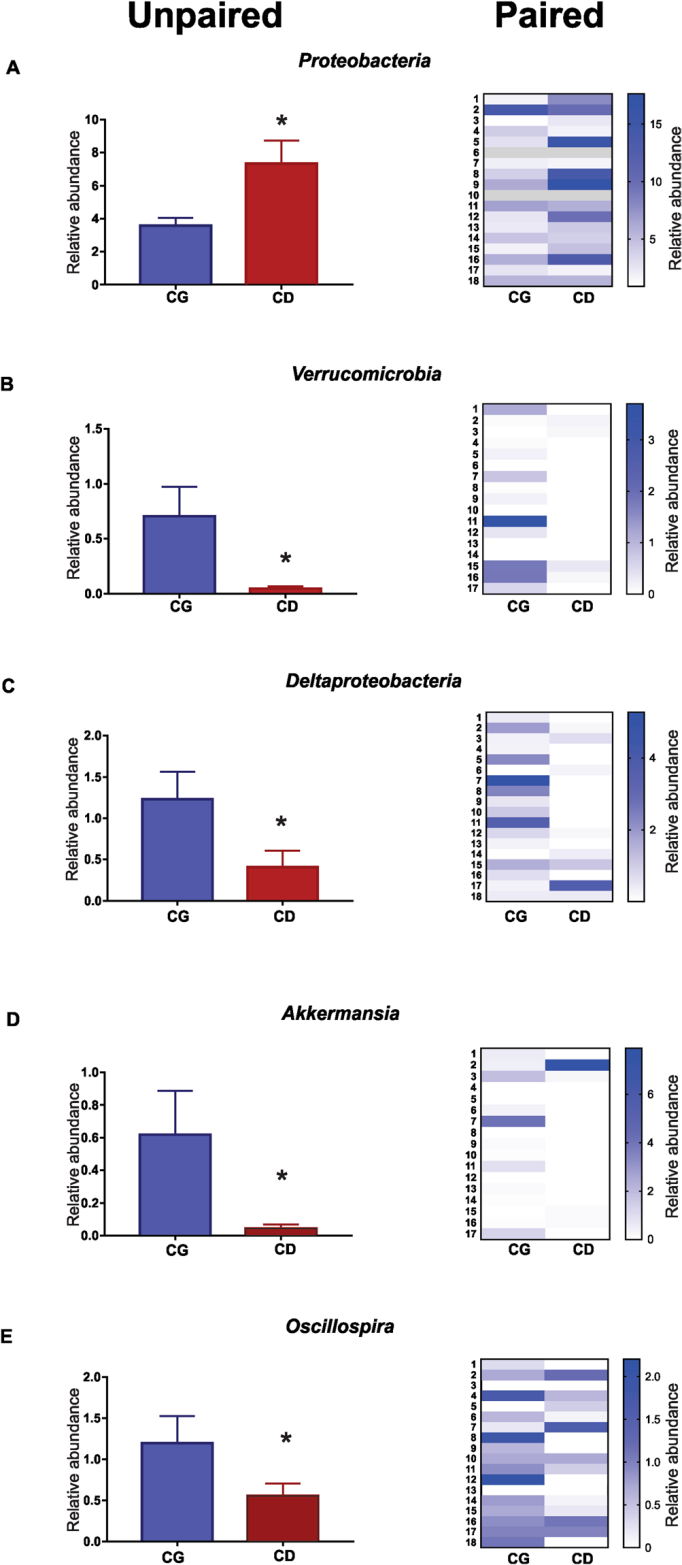
Comparison (paired and unpaired) relative abundance of between the CG and CD groups. (**A**) *Proteobaceria* (CG 3.5% ± 0.47 *vs* CD 7.4% ± 1.4; *p = 0.037 unpaired test and p = 0.016 paired test). (**B**) *Verrucomicrobia* (CG 0.40% ± 0.19 *vs* CD 0.02% ± 0.02; *p = 0,014 unpaired test and p = 0.003 paired test). (**C**) *Deltaproteobacteria* (CG 1.23% ± 0.33 *vs* CD 0.4% ± 0.21; *p = 0.0006 unpaired test and p = 0.007 paired test). (**D**) *Akkermansia* (CG 0.68% ± 0.27 *vs* CD 0.04% ± 0.02; p = 0.002 unpaired test and p = 0.005 paired test). (**E**) *Oscillospira* (CG 1.20% ± 0.33 *vs* CD 0.55% ± 0.15; *p = 0.024 unpaired test and p = 0.025 paired test).

In addition, α diversity among all samples were compared, therefore CD showed more species diversity variability profile than CG, based on Shannon index (Fig. 2). In general, the individuals residing in different houses (CG) group have a range Shannon index (approximately 3.2 up to 4) while CD group showed a range Shannon index (approximately 1.8 up to 4.6) (Fig. 2). Patients with CD showed a lower microbial α diversity compared with the CG as reflected by Shannon indexes (p = 0.047). Regarding α diversity, between groups, it was observed that the control group (CG) have a very similar alpha diversity while CD group do not show this homogeneity (Fig. 2B). The analysis of the gut microbiota showed no significant change in the proportion of the two most abundant microbial phyla: *Firmicutes* and *Bacteroidetes* between CD and CG groups (Fig. 3). Contrarily, the CD group showed greater abundance of the phylum *Proteobacteria* (CG 3.5% ± 0.47 *vs* CD 7.4% ± 1.4; p = 0.037 unpaired test and p = 0.016 paired test) (Fig. 4A) and lower of the phylum *Verrucomicrobia* (CG 0.40% ± 0.19 *vs* CD 0.02% ± 0.02; p = 0.014 unpaired test and p = 0.003 paired test) (Fig. 4B).

*Deltaproteobacteria* class, which contains most of the Sulfate-reducing bacteria, was reduced in the CD group. The relative abundance of *Deltaproteobacteria* class was high in CG group compared with CD (CG 1.23% ± 0.33 *vs* CD 0.4% ± 0.21; p = 0.0006 unpaired test and p = 0.007 paired test) (Fig. 4C). In addition, there was reduction of the beneficial genera *Akkermansia* (CG 0.68% ± 0.27 *vs* CD 0.04% ± 0.02; p = 0.002 unpaired test and p = 0.005 paired test) (Fig. 4D) and *Oscillospira* (CG 1.20% ± 0.33 *vs* CD 0.55% ± 0.15; p = 0.024 unpaired test and p = 0.025 paired test) (Fig. 4E) when compared to CG^19^. Although the analysis of the gut microbiota in this study showed a reduction in the genera *Dialister and Bifidobacterium* in the CD group (Supplementary Figure 1S), we did not find significant changes in the proportion of the species that have already been correlated with active CD like *Bifidobacterium adolescents, Dialister invisus and Faecalibacterium prausnitzii*^20^.

### Saccharomyces cerevisiae quantification

**Figure 5:**
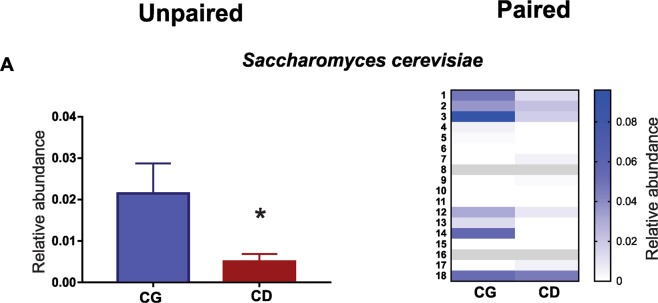
Quantification of Saccharomyces cerevisiae by qPCR. Comparison of *Saccharomyces cerevisiae* quantification between CD group and CG group (CG 0.02% ± 0.007 *vs* CD 0.005% ± 0.002 *p = 0.01 unpaired test and p = 0.008 paired test).

*Saccharomyces cerevisiae* may exhibit a protective role in the inflammatory process. Our data showed that the proportion of *Saccharomyces cerevisiae* was significantly lower in CD group when compared with CG group (CG 0.02% ± 0.007 *vs* CD 0.005% ± 0.002 p = 0.01 unpaired test and p = 0.008 paired test) (Fig. 5) by quantification using qPCR.

**Figure 6:**
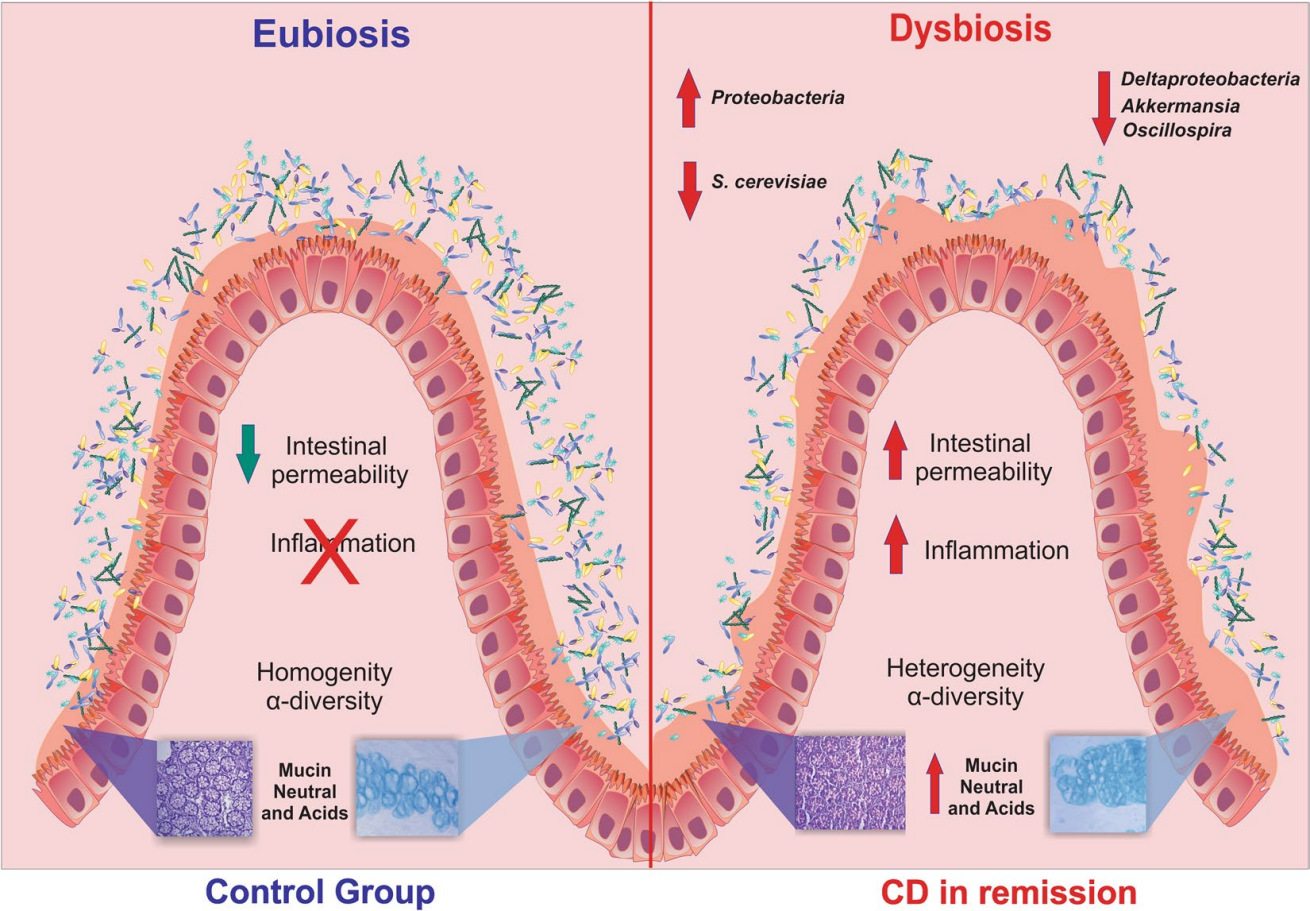
Graph abstract. CD patients, in remission disease, presented an increase of inflammation and intestinal permeability. In addition, CD patients also presented an increase in the amount of the neutral and acid mucins that is associated with a globally disturbed microbiota, with a reduction of the class *Deltaproteobacteria*, the genera *Oscillospira* and *Akkermansia*, and *S. cerevisiae*.

The significant findings of this study are summarized in Fig. 6.

## Discussion

The results of the present study demonstrated that the GI tract of CD patients in remission still display dysbiosis, that is characterized by reduced microorganism diversity. Additionally, when compared to the control group, which resided in the same home, there was an observed increase in the amount of *Proteobacteria* and a decrease in *Verrucomicrobia*. The CD group also presented increased mucins production.

According to Rothschild *et al*.^21^, individuals that experience the same environmental factors tend to have a similar gut microbiota compositions. Additionally, it is known that microbiota vary according to geography, cultural habits, age, and lifestyle factors that include diet, smoking, physical activity as well as others. Thus, in order to avoid potential biases when comparing CD patients to control group all of the study subjects must be exposed to the same environment^8,22–24^. In the present study, our control group consisted of people who were considered healthy, resided in the same home, had a similar hygiene status, consumed identical diets, and were, in general, exposed to equivalent environmental and other common lifestyle factors that could influence the composition of the gut microbiota.

The inflammation caused by CD, frequently results in damage to the intestinal wall^7^. The colonic mucus barrier provides a protective layer against potential antigens, pathogenic bacteria and metabolites produced by microorganisms present in the intestinal lumen. Additionally, the goblet cells produce mucins, which are connected by disulfide bridges and impede bacteria from penetrating the intestinal lumen^6^. It is important to mention that individuals with CD produce higher levels of mucins, that could attenuate the inflammatory response. Interestingly, the results herein showed that, even during remission CD patients also exhibit augmented mucin production, despite most of these patients not displaying any signs of inflammation, as revealed through colonoscopies.

The novelty of our study comes from integrating the gut microbiota composition and mucin production and evaluating how these changes influence the pathology of CD during remission. In this sense, it is tempting to speculate that the modulation of the gut microbiota may stimulate mucin production. In fact, we observed that the *Oscillospira* and *Akkermansia* genera were reduced in the CD group, when compared to the controls. Interestingly, the bacteria belonging to these two genera have been associated with the degradation of mucins and reductions in the proportions of these genera result in increased intestinal permeability^25,26^. Moreover, both neutral and acid mucin concentrations were increased in the CD group during remission.

With regards to other types of microorganisms, sulfate-reducing bacteria (SRB) obtain their energy through the reduction of sulfate to hydrogen sulfide (H_2_S). The SRB produced H_2_S can induce cytotoxity in human intestinal epithelial cells, promoting cellular damage and even cell death^25,27^. Most SRB are members of the *Desulfovibrio* genus in the *Deltaproteobacteria* class, and were found to be reduced in the CD group (p = 0.0166) of the present study.

The reduction in the proportion of the *Oscollospira*; *Akkermansia* genera and SRBs detected in the CD group during remission, may account for the increased mucin production, and may represent a compensatory mechanism for handling the CD-mediated inflammation. The CD group during remission presented a different gut microbiota when compared to both the healthy subjects (HS) and patients with active CD, apparently representing an intermediate microbiota composition.

In addition, a previous study reported that CD patients also have higher proportions of fungi in their intestines, with increased *C. albicans* and decreased *S. cerevisiae* content^28^. Herein we showed that CD patients in remission have reduced *S. cerevisiae* levels when compared with the control group. This result is similar to what has been observed in patients with active CD^29^. This is also relevant to inflammation, since a previous study with mice demonstrated that *S. cerevisiae* supplementation promoted a significant reduction in TNF-α expression and an increase in IL-10 production^30^. Through this anti-inflammatory effect *S. cerevisiae* can contribute to the regulation of the inflammatory process in patients with CD^29^, and its reduced presence may make these individuals more susceptible to relapses.

Our study has some limitations. First, it is a cross-sectional study and these results are observational. Secondly, we have not studied patients with active CD. Thirdly, the fungal analysis was limited to just one species (*S. cerevisiae)*.

In conclusion, during remission CD patients present increased amounts of the neutral and acid mucins that are accompanied by a global dysbiosis. In particular, we identified a reduction in the *Deltaproteobacteria* class, *Oscillospira* and *Akkermansia* genera, and *S. cerevisiae*. While these results are encouraging future studies need to further investigate the relationship between the gut microbiota, and mucin production in CD. Furthermore, studies focusing on patients with active CD, CD in remission and healthy subjects need to be undertaken to further validate the results presented here.

## Methods

### Study design and population

We did an analytical cross-sectional single center study (IBD clinics of Campinas State University – Unicamp – Brazil), with CD patients and healthy controls. Inclusion criteria were CD patients with diagnosis confirmed by means of clinical, endoscopic and histological criteria and for control group adults residents in the same house, without previous history of chronic disease. Exclusion criteria included individuals that used antibiotics or probiotics during the previous 2 months. Disease activity in CD patients was assessed by the CDAI score^31^ and endoscopic findings. CDAI score under 150 were considered inactive disease (clinical remission).

Clinical data, disease classification, medications and comorbidities were collected on the same day of colonoscopy procedure. Fecal samples were collected by the subjects at home using Sarstedt tubes (Sarstedt, Nümbrecht, Germany) filled with a preservative buffer and brought to the IBD clinics within 24 hours after defecation. Stool samples were stored in frozen at -80 °C for microbiota analyses.

### Metagenome profile

Total DNA of fecal samples was extracted with the Stool PSP Spin DNA kit (STRATEC Biomedical AG, Germany), an integrated system for collecting, transporting and storing feces samples and subsequent DNA purification.

To profiling microbiota composition, the hyper-variable region (V3-V4) of the bacterial 16S rRNA gene was amplified by following the Illumina *16S Metagenomic Sequencing Library Preparation guide*^32^ which uses the following sequence: 338F - 5′-TCGTCGGCAGCGTCAG ATGTGTATAAGAGACAGCCTACGGGNGGCWGCAG -3 and 785R - 5′-GTCTCGTGGGCTCGGAGATGTGTATAAGAGACAGGACTACHVGGGTATCTAATCC-3′ (2 × 300 bp paired‐end an insert size of ~550 bp).

### Bioinformatic and statistical data analysis

The fastq sequences were analysed using DADA2 tool as describe by Callahan *et al*.^33^, that allows to recover single-nucleotide resolved Amplicon Sequence Variants (ASVs) from amplicon data. The default parameters were used to improve the overall quality of the sequences, the reads were filtered and trimmed using the “filterAndTrim” function implemented in DADA2 as described in https://benjjneb.github.io/dada2/tutorial.html. Low quality bases at the end of the reads were removed and the truncLen option was set to 280 and 220 to trim the forward and reverse fastq files respectively. Moreover, the sequences were also trimmed at the 5′ end using the trimLeft option set to 17 and 21 for the forward and reverse reads respectively. The taxonomic assignment was subsequently performed using the naïve Bayesian classifier method implemented in DADA2 using as reference the SILVA database. Finally, the final phylogenetic tree of the ASVs was obtained using the function AlignSeq implemented in DECIPHER^34^ R package to create the multiple sequence alignment and the Fast Tree program^35^.

Statistical analysis was performed on R (Version 3.4.4) using the following R packages: phyloseq (version 1.24.0) to facilitate the import, storage, analysis, and graphical display of microbiome census data^36^. Data were pre-processed filtering features with less than 10 read counts and present in less than 2 samples^36^. Vegan (version 2.4.2) for PERMANOVA analysis^37^. Shannon diversity indices and bar plot graphical were generated by using the R package ggplot2. The longitudinal microbiome studies was carried out from q2-longitudinal, a software plugin for the QIIME 2 microbiome analysis platform (https://qiime2.org)^38^.

### Staining techniques for neutral and acid mucins

The biopsies of large bowel mucosa content of the neutral and acid mucins were determined individually to modify histochemical Periodic Acid Schiff (PAS)^39^ and Alcian Blue (AB) techniques. The slides were read under an ordinary optical microscope with a final magnification of 200×. The histological parameters were analyzed qualitatively and quantitatively by a pathologist with experience of diseases, of the digestive tract who was unaware of the origin of the material and the objectives of the study. The neutral mucins stained magenta, while the acid mucins stained blue.

### Image processing, computer-assisted

The images selected were captured on a video camera that had been coupled to an optical microscope. These images were processes and analyzed using the NIS-Elements (Nikon Corporation. Instruments Company, Japan) software, installed in a computer with good image processing capacity. By means of colored histograms in RGB system (Red, Green, Blue) the software determined the color intensity in number of pixels in each field selected and transformed the final data into percentage expressions by analyzed fields. The final value in the segments with and without intestinal transit was the mean of the values found from evaluating three different fields.

### Quantification of *Saccharomyces cerevisiae* by qPCR

For quantification of *Saccharomyces cerevisiae* the qPCR was performed using the primers and probe described by Mallant-Hent *et al*.^40^. Briefly, reaction was performed in 12 μl total volume containing 1x Universal Master Mix (Applied Biosystems), 150 nmol of both primers (5′-GAA ATG CCA CCG TGA ATG C and 5′-CTT TGG TGG TGA TCC TCT ATG ATT G), 100 nmol of the probe (FAM-TGG CAC CAT GAA CCC TAG CGT CGT T-TAMRA), and 120 ng of DNA extracted from stool samples. This reaction was performed on the QuantStudio 6 Flex Real-Time PCR System (Applied Biosystems - Life Technologies Corp., USA). For the quantification, a standard curve was performed with *Saccharomyces cerevisiae* (strain was kindly donated by Laboratory of Enzymology and Molecular Biology of Microorganisms/State university of Campinas).

### Statistical analyses

#### Sample size

based on pilot study data, the sample size calculation was done having as the main variable the relative contribution of *Proteobacteria* percentage. Considering the paired group (pilot with 6 subjects in each group, effect size = 0.90), assuming α in 5% and β in 5% (power 95%) 12 subjects were necessary in each group. The Software used for the calculation sample size was G*Power version 3.1.2. (Program written, concept and design by Franz, Universitat Kiel, Germany, freely available windows application software)^41^.

The inclusion of the CD patients versus control groups were expressed as medians and percentiles (interquartile range (IQR), 25–75%) for continuous variables and as frequency for categorical variables. For the qualitative variables, the Fischer exact test and the chi-square test (χ^2^) were select. The Mann-Whitney U-test (non-parametric distribution) was used to compare continuous variables between categories. The significance level adopted was 5% for all statistical tests (*p*-value < 0.05). Statistical analyses were used according to SSPS v.20.0 software (IBM Inc., Armonk, NY, USA).

### Ethical statement

During routine visits, subjects who agreed in participating in the study signed up an informed consent form. All methods were performed in accordance with the relevant guidelines and regulations. The study was approved by Institutional Ethics Review Board at Unicamp, in Campinas, Brazil, under reference number 885.749/14.

## Supplementary information

Supplementary INFO
