# Supplementary material for: Remission in Crohn’s disease is accompanied by alterations in the gut microbiota and mucins production
Source: Sci Rep. 2019 Sep 13;9:13263. doi: 10.1038/s41598-019-49893-5 (PMC6744406; doi:10.1038/s41598-019-49893-5)
Supplement: Supplementary file 1 — Supplementary INFO [file 41598_2019_49893_MOESM1_ESM.pdf]

Remission in Crohn's disease is accompanied by alterations in the gut microbiota and mucins production.

Daniéla Oliveira Magro[2] PhD[#] danimagro@terra.com.br

Andrey Santos[1] PhD*[#] andreysts@gmail.com

Dioze Guadagnini[1] MD diozeg@gmail.com

Flavia Moreira de Godoy[3] flagodoy94@gmail.com

Sylvia Helena Monteiro Silva[1] sylmonteiro3@gmail.com

Wilson José Fernandes Lemos [4] juniorjflemos@gmail.com

Nicola Vitulo [4] nicola.vitulo@univr.it

Sandra Torriani[4] sandra.torriani@univr.it

Lilian Vital Pinheiro[2] MD lilian_vp@yhaoo.com.br

Carlos Augusto Real Martinez[2]; PhD carmartinez@uol.com.br

Mario José Abdalla Saad[1] MD; PhD msaad@fcm.unicamp.br

Claudio Saddy Rodrigues Coy[2], MD, PhD claudiocoy@gmail.com

Table 1S. PERMANOVA on CD and CG dataset: Permutation test for Adonis under reduced model, Permutation: free, Number of permutations: 10000,

	Df	SumOfSqs	R2	F	Pr(>F)
Origin	1	0.2602	0.04627	1.6439	0.023 *
COLONO*	5	0.7737	0.13756	0.9775	0.4996
Residual	29	4.5906	0.81618		
Total	35	5.6245	1		

Significance codes: 0 '***' 0.001 '**' 0.01 '*' 0.05 '.' 0.1 ' ' 1.

*Disease activity in CD patients was assessed by the CDAI score and endoscopic findings

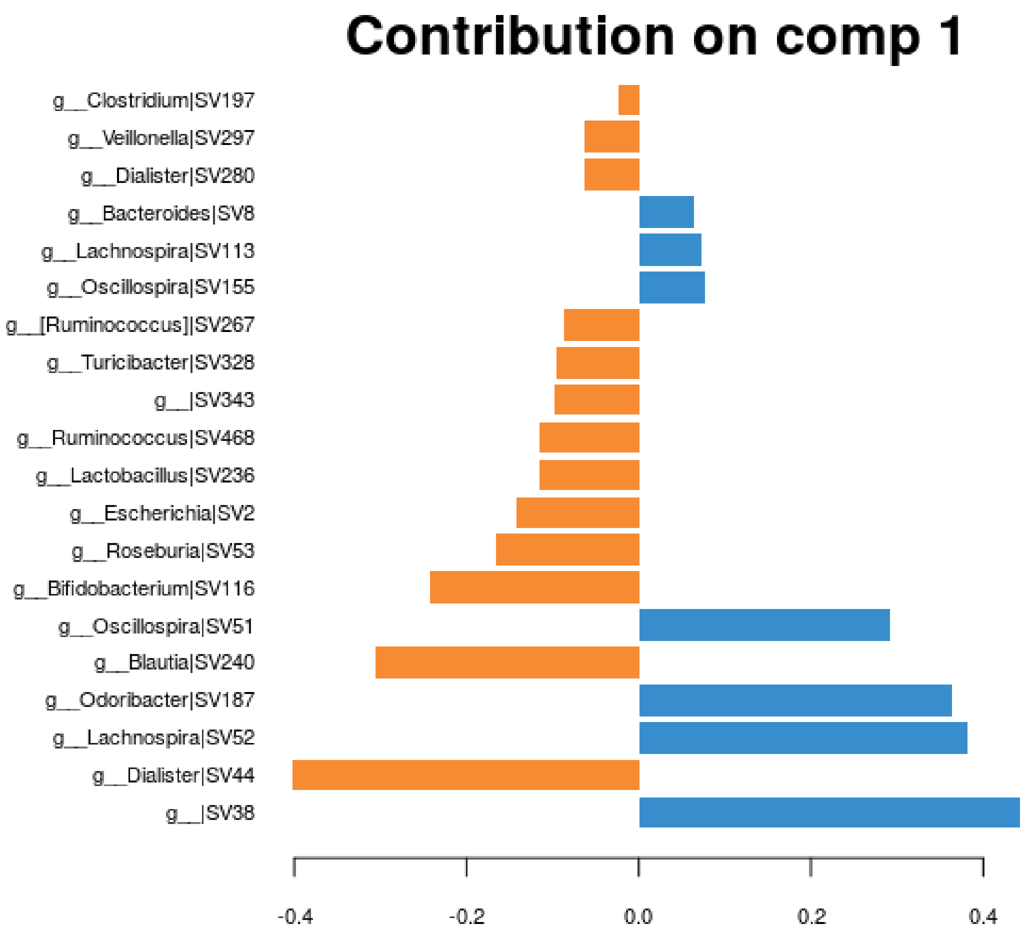

Figure 1S Genera differentiating between the CG and CD. Contribution of each genus identified by sPLS-DA, samples origin: CG (blue) or CD (orange).
